# 

**Published:** 1894-03-10

**Authors:** 


**Notice to Advertisers and Subscribers.**

All Advertisements, Orders for Copies of The Hospital, and Business Communications should be addressed to The Publisher, *not to the Editor*, The Hospital, 428, Strand, W.C.

The Cost of Subscription, post free, is as follows:—Per week, 2½d.; per quarter, 2s. 8d.; per half-year, 5s. 3d.; per annum, 10s. 6d. Foreign:—Per Annum, 12s. 8d.; payable, in all cases, in advance.

**OUR NEW OFFICES.**

428, Strand, W.C., will henceforth be the Office of this Journal.

**Notices of Births, Deaths, and Marriages** are inserted in this column at a charge of 2s. 6d. for an announcement not exceeding four lines.

### NOTICE TO CORRESPONDENTS.

All MS., Letters, Books for Review, and other matters intended for the Editor should be addressed The Editor, The Lodge, Porchester Square, London, W.

The Editor cannot undertake to return rejected MS., even when accompanied by stamped directed envelope.

**"Burdett's Hospital Annual," 1894.**

Fifth year of publication. 700 pp. Boards, gilt lettering. A most complete guide to British, Colonial, Indian, and American Hospitals, Dispensaries, Nursing and Convalescent Institutions, and Asylums. Price 5s. Published by The Scientific Press (Limited), 428, Strand, W.C.

**NOTICE.—The Index for Vol. XIV. (ending Sept. 30th, 1893) is on sale at the Office of this Journal, price 2½d., post free.**

### Scraps and Gleanings.

A proposal for an infectious diseases hospital is being pressed upon the Plomesgate Guardians.

Six hundred and ninety-seven patients were under treatment at the Royal Ear Hospital during February.

The Cheyne Hospital for Sick and Incurable Children, Cheyne Walk, Chelsea, has received a grant of £25 from the Grocers' Company.

The financial statement of the Samaritan Hospital, Ulster, for the year shows a balance of £167 in favour of the institution.

We are requested to state that on and after March 10th, *The Pioneer of Fashion* will be published under the title of *The Court and Pioneer of Fashion*, with many new features and attractions.

The National Children's Hospital, Harcourt Street, Dublin, celebrated its annual meeting recently. The position attained by this hospital, and its satisfactory balance-sheet were duly reported.

The treasurer's report at the annual meeting of the Ashburton and Buckfastleigh Cottage Hospital states that the receipts during the year have been £222, and that the expenditure has been £250.

There have been 613 patients received at the Birkenhead Borough Hospital during the last twelve months. In spite of special efforts by the committee the debit balance has increased during this period from £281 to £478.

The latest report of Barnard Castle Dispensary, Darlington, shows another year's good work to be added to the list of successes already recorded. A balance is left in hand towards the working expenses of the institution.

The annual general meeting of the Governors of Forres Leanchoil Hospital took place recently. The funds at the close of the accounts ending December 31st, 1892, show that the balance of income over expenditure is £7.

The Governors of the Glasgow Royal Infirmary in their late report on that charity, state that "if the infirmary is to be conducted as hitherto, it is indispensable that not less than £10,000 be added to the annual subscription."

The German Surgical Congress will be held in Berlin on April 18th to 21st. The German Congress of Internal Medicine has been postponed to 1895, owing to difficulties arising from the International Medical Congress in Rome.

The income for the Grantham Hospital for the year has been £1,323 which shows an increase of £21 on last year's receipts. The expenditure for the period under review has been £1,859, which is a sum over £500 in excess of the receipts.

Supporters and friends of the Tavistock Cottage Hospital and Dispensary held their annual meeting recently. The chairman reported a decided progress in the usefulness of the institution. A balance of £44 is carried forward to the next account.

At the annual report of the Convalescent Home, Stillorgan it was stated that no death has occurred in the institution during the year under review. The income from all sources towards this most useful charity is £1,500, which is an insufficient one for its maintenance.

The County Council have again the insanitary condition of the Regent's Canal under consideration. It will be a clever county council indeed which formulates regulations not too ruinously expensive which will render the canal aught but little better than an open drain.

The annual meeting of the friends and supporters of the Liverpool Hahnemann Hospital and Homœopathic Dispensaries was held lately. This is the sixth year of activity of the hospital, and fifty-second of the dispensary. The treasurer reports a deficit of £700 on the finances.

The annual meeting of the Longmore Hospital for Incurables took place recently. The annual subscriptions toward which institute show an increase from £1,61[illegible] in 1892, to £1,923 last year; the donations also have risen. The total number of patients in the hospital on December 31st was 83.

The balance sheet of the Newton Cottage Hospital and Dispensary, at the annual meeting just taken place, shows that the subscriptions, donations, collections, legacies, &c., during the year, amounted to £2,400, and the expenditure to £2,500, thus leaving an adverse balance on the accounts.

One reads funny things in the reports of hospitals occasionally. A certain hospital in the eastern counties expresses regrets that receipts from "the sale of dripping and patients' washing are smaller than last year." To the casual reader this sentence is rather ambiguous, and not quite pleasant.

The sixth annual report of the Leaf Homœopathic Hospital, Sussex, shows a considerable increase in the total number of patients treated during that period. It is highly satisfactory to the management to find that old patients and their friends have collected £11 during the year towards the funds of the institution.

The directors' report of the Glasgow Convalescent Home stated that during the year 1,644 patients had been admitted to the home. The total expenditure for the year in the annual revenue accounts exclusive of the extraordinary expenditure, has been £2,10[illegible]. A general increase in the subscriptions has been reported.

The annual court of governors of the Bradford Children's Hospital was held recently. The report announces a further increase in the amount of beneficent work accomplished by the institution. There were 333 patients treated during the year under review. The finances of the hospital are not in a very flourishing condition.

During the last twelve months 85 patients have been treated at the Victoria Cottage Hospital, Guernsey. The treasurer's accounts show that the expenditure has exceeded the receipts by £23. A new ward has been opened called "The Elizabeth Mollet Ward," the funds for which was collected from friends, and from those who benefited by Miss Mollet's instructions in ambulance work.

The twenty-third annual meeting of governors of the Harrowgate Cottage Hospital was recently held. In connection with the proposed extention of this institution, the committee announced that during the year they had received a munificent donation of £500 from the Misses Wardell, of Harrowgate, and this sum being unfettered by any condition, it will be set aside for the building fund.

### BOOKS RECEIVED.

MESSRS. FISHER UNWIN.

"Mrs. Thorndale's Cousin." By E. M. Bacot.

"Normal Labour, as conducted in the Frauen Universität Klinik, Berlin." By Moffat Flynn, F.R.C.S.

DIGBY LONG AND CO.

"Maria Countess of Saletto." Translated from the Italian of E. Arbib. By Sydney King.

"Marie Charlotte de Corday." A Centenary Monograph. By Mary Jeaffreson.

**Periodicals and Pamphlets.**—Birmingham Medical Review, the Therapist, Charity Organisation Review, Commerce, the Christian Commonwealth, Amateur Gardening, London, Reich's Medicinal Anzeiger, Review of the Churches; Friendly Greetings; Ann Judson; Our Little Dots; Child's Companion; Light in the Home; the Cottager and Artisan; the Sunday at Home; the Leisure Hour; the Girl's Own Paper; the Boy's Own Paper; the Cornhill Magazine; St. Nicholas; Service for the King; the Zenana; the Planter; Cassell's Magazine; Inserenten Zeitung; the Humanitarian; Guy's Hospital Gazette; Child's Guardian.

### Medical Vacancies and Appointments.

### VACANCIES.

### Resident Medical Officers.

Bradford Children's Hospital. House Surgeon (to dispense); doubly qualified. Salary £70, with board, residence, and washing. Applications and testimonials to C. V. Woodcock, Secretary, by March 12th.

Coombe Lying-in Hospital, Dublin. Assistant Master. Tenure of office three years; premium to Master £200. Applications and testimonials to the Master, Coombe Hospital, Dublin.

County Asylum, Prestwich, Manchester. Assistant Medical Officer; unmarried. Salary commencing at £100 per annum, increasing to £200 by successive yearly increments of £25, with apartments, board, attendance, and washing. Applications to the Superintendent.

Joint Counties Lunatic Asylum, Carmarthen. Medical Superintendent. Salary £500 per annum, with unfurnished house, garden, produce, fire, light, and washing. Applications and testimonials to be forwarded to W. Morgan Griffiths, Solicitor, Carmarthen, by March 24th.

Miller Hospital and Royal Kent Dispensary, Greenwich Road, S.E. Junior Resident Medical Officer. The post is tenable for six months, with prospect of re-election for the same period. Salary £30 per annum, with board, attendance, and washing. Applications and testimonials to Major-General G. R. Roberts, Honorary Secretary, by March 10th.

Roxburgh District Asylum, Melrose, N.B. Assistant Medical Officer. Salary £100 per annum, with board, lodging, and washing. Applications and testimonials to Dr. Carlyle Johnstone, Medical Superintendent.

Royal Hospital for Diseases of the Chest, City Road, E.C. House Physician. Appointment for six months. Salary at the rate of £40 per annum, with board and lodging. Applications and testimonials to the Secretary by March 14th.

Royal South Hants Infirmary, Southampton. House Surgeon. Salary £100 per annum, with board and lodging. Applications with testimonials to the Secretary, T. A. Fisher-Hall, by March 10th.

Royal Surrey County Hospital, Guildford. House Surgeon. Salary £80 per annum, with board, lodging, and laundry. Applications to the Honorary Secretary by March 10th.

Tunbridge Wells General Hospital. Resident House Surgeon, also to act as Secretary. Must be unmarried. Salary £100 per annum, with board, furnished apartments, gas, firing, and attendance. Applications and testimonials to Henry Harris, Assistant Secretary, at the General Hospital, by March 13th.

### Non-Resident Medical Officers.

County of Northumberland. Medical Officer of Health. Salary £500 per annum, with travelling expenses. Appointment for three years. Applications and testimonials, endorsed "Medical Officer," to C. D. Forster, Clerk to the Council, by March 24th.

Dental Hospital of London, Leicester Square, W.C. Assistant Dental Surgeon. Applications to J. Francis Pink, Secretary, by March 12th.

Hospital for Sick Children, Great Ormond Street, Bloomsbury, W.C. House Surgeon to Out-patients. Appointment for six months, but the holder will be eligible for a second term of office. Salary 25 guineas. Applications and testimonials to Adrian Hope, Secretary, by March 20th.

### The Hospital Advertiser.

### MEDICAL APPOINTMENTS.

ROYAL SURREY COUNTY HOSPITAL, GUILDFORD.

WANTED, an ASSISTANT HOUSE SURGEON. Board, Residence, and Laundry found. No salary.

Applications, with copies of testimonials, to be sent to the Hon. Secretary, Royal Surrey County Hospital, Guildford, not later than March 10th next. (1,193)

THE HOSPITAL FOR SICK CHILDREN, GREAT ORMOND STREET, BLOOMSBURY, W.C.

